# Intersectoral Action for Addressing NCDs through the Food Environment: An Analysis of NCD Framing in Global Policies and Its Relevance for the African Context

**DOI:** 10.3390/ijerph182111246

**Published:** 2021-10-26

**Authors:** Amy Weimann, Maylene Shung-King, Nicole McCreedy, Lambed Tatah, Clarisse Mapa-Tassou, Trish Muzenda, Ishtar Govia, Vincent Were, Tolu Oni

**Affiliations:** 1 Research Initiative for Cities Health and Equity (RICHE), Division of Public Health Medicine, School of Public Health and Family Medicine, University of Cape Town, Cape Town 7925, South Africa; trishmuzenda@gmail.com (T.M.); Tolullah.Oni@mrc-epid.cam.ac.uk (T.O.); 2 African Centre for Cities, University of Cape Town, Cape Town 7701, South Africa; 3 School of Public Health and Family Medicine, University of Cape Town, Cape Town 7925, South Africa; maylene.shungking@uct.ac.za (M.S.-K.); MCCNIC003@myuct.ac.za (N.M.); 4 Health of Populations in Transition Research Group (HoPiT), University of Yaoundé I, Yaoundé 8046, Cameroon; Lambed.Tatah@mrc-epid.cam.ac.uk (L.T.); mapatassou@yahoo.fr (C.M.-T.); 5 Global Diet and Physical Activity Research Group, Medical Research Council Epidemiology Unit, University of Cambridge, Cambridge CB2 0QQ, UK; 6 Caribbean Institute for Health Research, The University of the West Indies, Mona Kingston 7, Jamaica; ishtargovia@gmail.com; 7 Center for Global Health Research, Kenya Medical Research Institute (KEMRI), P.O. Box 1578, Kisumu 40100, Kenya; vincentwere@gmail.com

**Keywords:** healthy policy, noncommunicable diseases, NCDs, policy analysis, diet, nutrition, intersectoral, Africa

## Abstract

Noncommunicable diseases contribute the greatest to global mortality. Unhealthy diet—a prominent risk factor—is intricately linked to urban built and food environments and requires intersectoral efforts to address. Framings of the noncommunicable disease problem and proposed solutions within global and African regional diet-related policy documents can reveal how amenable the policy landscape is for supporting intersectoral action for health in low-income to middle-income countries. This study applied a document analysis approach to undertake policy analysis on global and African regional policies related to noncommunicable disease and diet. A total of 62 global and 29 African regional policy documents were analysed. Three problem frames relating to noncommunicable disease and diet were identified at the global and regional level, namely evidence-based, development, and socioeconomic frames. Health promotion, intersectoral and multisectoral action, and evidence-based monitoring and assessment underpinned proposed interventions to improve education and awareness, support structural changes, and improve disease surveillance and monitoring. African policies insufficiently considered associations between food security and noncommunicable disease. In order to effectively address the noncommunicable disease burden, a paradigm shift from ‘health for development’ to ‘development for health’ is required across non-health sectors. Noncommunicable disease considerations should be included within African food security agendas, using malnutrition as a possible intermediary concept to motivate intersectoral action to improve access to nutritious food in African low-income to middle-income countries.

## 1. Introduction

The formulation of policy, including the manner issues are secured onto agendas, is incredibly complex and messy [1]. Multiple factors including politics, context, and a variety of actors determine whether issues are elevated into a ‘problem status’ and accepted onto agendas and influence how policy goals and objectives are formulated and how policies are implemented [2,3,4]. In this complex process, formal health policy is generally referred to as the recorded decisions, plans, and commitments that governments and actors make under the influence of broader concepts of power and process for the purpose of meeting specific health-related objectives for improving the health of populations [5,6]. These are developed at various levels, such as from global to local.

Formulating policies within one sector is complex, and when issues transcend sector boundaries and ideally require intersectoral policy action, it becomes even more complicated. Addressing noncommunicable diseases (NCDs) and their determinants (including unhealthy diet which forms one of the major risk factors alongside alcohol, tobacco use, and inadequate physical activity) is one such issue that requires a complex set of policy considerations and engagements from multiple sectors including agriculture, education, health, and the private sector [7,8]. The World Health Organization’s (WHO) [9] Health-in-All-Policies approach (HiAP) advocates for intersectoral action that considers health implications of decision making in order to address key health outcomes and their risk factors. The HiAP approach has been adopted by many high-income countries, yet it has not been systematically implemented in Africa—a region in which many low-income to middle-income countries (LMICs) are facing a growing burden of NCDs alongside existing burdens of communicable diseases and malnutrition [10,11]. Furthermore, it remains unclear if African regional policies are expressed in a manner that makes them amenable for supporting increased intersectoral action for addressing NCDs.

### Conceptual Framework

Various theories and frameworks largely focusing on understanding the policy process and how certain issues make it onto policy agendas include Walt and Gilson’s [4] policy triangle framework; Shiffman and Smith’s [12] policy prioritisation framework; and Kingdon’s [3] theory of multiple streams of policymaking, which highlights complex interactions between problems, policies, and political environments [3,13]. Specifically looking at Kingdon’s [3] theory, the ‘problem’ stream refers to key risks or challenges that policymakers feel demand policy attention and intervention [3,13]. The ‘policies’ stream relates to proposed policy actions that seek to address the problem, while the ‘politics’ stream refers to the political environment and whether it is conducive to supporting and building momentum for addressing the problem through the proposed solutions [3,13].

A second relevant body of work relates to frame theory. The way in which concepts and issues are presented and positioned through a particular worldview influences their uptake onto policy agendas and can inspire new policy directions and ways of thinking [14]. Policy framing, as an approach for exploring how an issue is presented and defined, has been widely used in analyses related to agenda setting [2,14,15]. By embedding ideas within key global concerns or political agendas, frames may shape general policy direction as well as public understanding and opinion on certain issues [2].

This study does not seek to explore agenda setting a priori. Rather, it draws on the idea of problem and policy streams from Kingdon’s [3] theory and in combination with framing theory and provides an alternative approach for exploring how the NCD-related ‘problem’ and ‘solution’ are expressed in policy and how amenable such policies are for supporting intersectoral action. Bacchi [16] (p. 10) states that “*some important contributions in the health policy field (and elsewhere) explicitly analyse how “problems” are conceptualized within policy documents*”, as opposed to the traditional approach of analysing how issues are framed for agenda-setting. An example of alternative policy framing was demonstrated by Smith et al. [17].

Framing contributes to certain policy challenges. Firstly, policy is socially constructed with a degree of cognitive bias lying at the root of policy decisions [2,18]. Secondly, the value placed on one-size-fits-all global approaches relative to addressing international issues such as the coronavirus 2019 disease pandemic, as opposed to unique context-specific local approaches, is argued to create ‘blind trust’ in the global system and, thus, can undervalue public needs in some contexts [18]. Lastly, Bennett, Glandon, and Rasanthan [19] caution that the framing of NCDs and their risk factors is likely to occur predominantly over wider urban environment or food system frames, which will require ambitious multisectoral and intersectoral interventions to address. Therefore, the frames through which NCD problems and solution approaches are currently presented will likely act as either barriers or facilitators for setting up further agenda for intersectoral action to address NCDs.

This study aimed to explore how global diet-related policies are reflected in regional (African) policies for the control of NCDs in LMICs by identifying key frames through which the NCD ‘problem’ and ‘solution’ are expressed within diet-related policies at the global level and to describe how these framings may be amenable for supporting intersectoral interventions for health, relevant to diet and food environments in the African context. Of note, views of policymakers could reveal additional insight into the interpretation of policy documents and the influence of wider policy contexts; however, this fell outside the scope of the study that sought to focus only on the analysis of policy document text. This analysis was conducted as part of wider retrospective policy analyses exploring the global, African regional, and selected national policy environments for the promotion of healthy diet interventions for addressing NCDs as part of The Global Diet and Activity Research Group and Network (GDAR) funded through the National Institute for Health Research Global Health Research initiative. The collective GDAR projects sought to research and address the burden of NCDs in LMICs. As part of a GDAR work package, this study built on wider global diet-related policy analysis work.

## 2. Materials and Methods

By applying a document analysis approach on the content of written policy documents, this retrospective policy analysis was conducted on global and African regional policies related to NCDs and diet.

### 2.1. Global Policy Documents

In February 2019, global diet-related policy documents relevant to NCDs from 2000 to 2019 were explored. An internet-based search was undertaken using Google Scholar™ in order to search websites of key United Nations (UN) agencies overseeing matters of health and diet or nutrition, namely the WHO and the Food and Agricultural Organization (FAO). The search approach sought to identify policy documents with either an explicit or implicit link to NCDs and which featured either an explicit or implicit link to diet or nutrition, food, salt, or sugar. Searches used the relevant primary term, abbreviations, key terms of interest, or alternative terms, as depicted in Table 1, in various combinations within search formulae. In all searches, documents up to the first 100 results were extracted for further data cleaning. This was a pragmatic decision made by the wider research group.

Documents were considered implicit if they did not clearly focus on NCDs or diet-themes, especially in their title, but contained sufficient subsidiary information relevant to NCDs and diet, for example, documents relating to obesity, malnutrition, and dental caries. Additional hand searches were conducted directly on the websites of the following organisations or agencies: The World Food Programme, the FAO, UN-Habitat, the World Bank, UNICEF, UN Healthy Cities, UN System Standing Committee on Nutrition, World Obesity, and the World Health Assembly. Additional snowballing occurred during analysis where sentinel documents were identified in reference lists.

**Figure 1:**
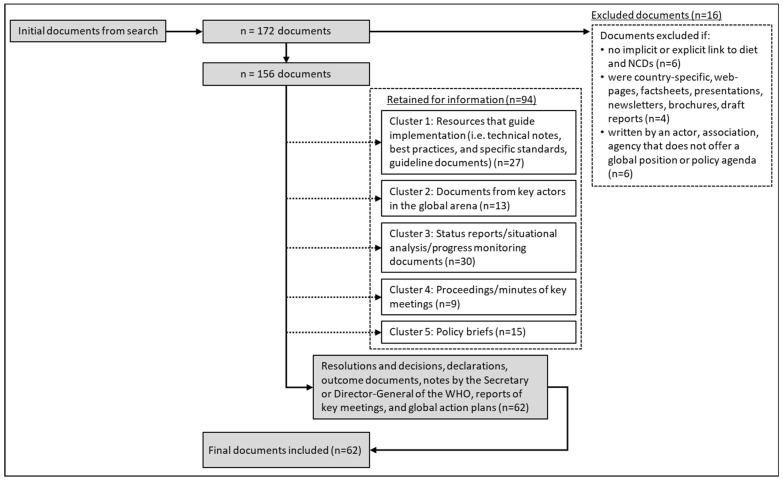
Flow diagram of data cleaning and document clustering process for the global policy analysis.

Figure 1 summarises the data cleaning process and the inclusion and exclusion criteria. Documents were considered for inclusion if they were written by an actor, association, or agency that provided a global position related to the topic of NCDs and/or diet, nutrition, salt, sugar, or food labelling. These included political declarations, resolutions, statements, and action plans. Documents were excluded if they did not have an implicit or explicit link to NCDs or an implicit or explicit link to diet. Supplementary policy documents such as policy briefs, guides, and reports were sorted into clusters and retained as background information, but they were excluded from analysis. The research team met regularly during the data collection phase in order to deliberate the inclusion and exclusion of documents.

A codebook was developed for the analysis. By using QSR International’s NVivo 12 Software [20], four policy documents were triple coded by three project researchers, and an additional two documents were then double coded so that 10% of the final document list had been coded by multiple researchers in order to ensure intercoder agreement. Initial phases of coding were conducted during a research team workshop so that any issues or queries could be discussed between researchers and addressed immediately. The coding process was iterative, and researchers maintained regular contact in order to ensure alignment.

A thematic analysis approach was conducted by three researchers. For the preliminary analysis, which occurred at a three-day deep-dive analysis retreat, two of the codebook nodes were analysed for key messages by each researcher. These key messages were presented, deliberated, and discussed in order to ensure all that researchers were comfortable with the thematic analysis method. The remaining NVivo nodes were analysed individually. Regular project team meetings and wider GDAR network meetings ensured that project timelines were adhered to, that the overall focus of the analysis stayed on course, and that opportunities were provided for critique or insights into the methodology, analysis, and synthesis of findings.

### 2.2. African Regional Policy Documents

Reflecting on the extensive and arduous data collection process for the global policy analysis, it was realised that most global policy documents were easily accessible from key organisation websites, such as the WHO and FAO. For the African level policy analysis, the websites of key regional organisations that were either directly or indirectly relevant to the context of NCDs and diet were extensively hand searched for policy documents from 2000 to 2019. Similar to the global level context, the regional data collection process sought to identify diet-related policy documents that were either explicitly or implicitly linked to NCDs and which featured either an explicit or implicit link to diet or nutrition, food, salt, or sugar. During the hand search, documents were considered if they had a clear focus on NCDs or diet-themes, especially in their title, or if they contained sufficient subsidiary information relevant to NCDs and diet. The process was open to snowballing using references within documents. In this context, policy documents were political declarations, resolutions, statements, policy agendas, action plans, and regional strategies.

The following key organisation websites were hand searched, which formed the initial collection of documents:

- World Health Organization: Regional Committee for Africa (WHO AFRO);
- New Partnership for Africa’s Development (NEPAD);
- African Development Bank Group;
- United Nations Population Fund (UNFPA);
- African Union Commission;
- Food and Agriculture Organization: Africa;
- World Food Programme;
- United Nations System Standing Committee on Nutrition.

**Figure 2:**
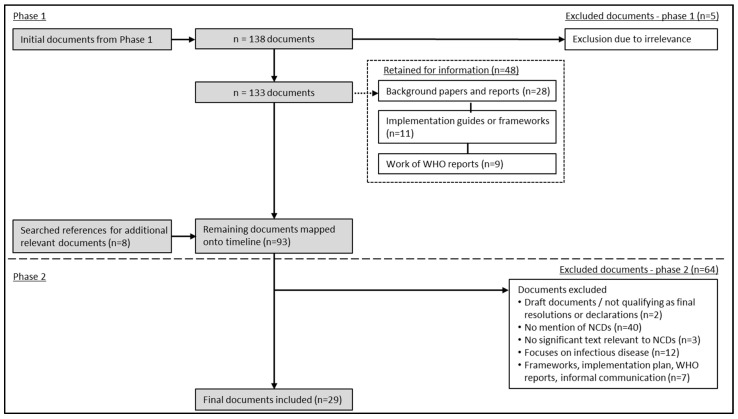
Flow diagram of search process of policy documents for the African region.

The global level data cleaning process was adapted for the African regional level which occurred over two phases, as illustrated in Figure 2.

Through Phase 1 data cleaning, five documents were excluded as they either related to a single African country context or focused on NCDs that fell outside the scope of this research study, e.g., sickle cell disease, cancer, and chronic respiratory disease. A further number of 48 documents were excluded from the core documents and retained for additional information as needed. Eight documents were further sourced by searching references. A total of 93 documents and key sentinel moments were identified and mapped onto a timeline for the African Region (see Table S1, available as a supplement). Phase 2 sought to refine the list of phase 1 policy documents in which 64 documents were further excluded. These included draft documents, which are those that primarily focused on infectious diseases and, therefore, only superficially mentioned NCDs, frameworks, implementation plans, and other informal communication which were included for interest in the timeline but were considered unnecessary for the framing analysis. In addition, documents were excluded if they did not mention NCDs within text, which was determined through an internal keyword search, as this study sought to explore policy problem and solution framing specific to NCDs.

Working off global policy analysis, a codebook was developed for regional policy analysis with codes aligned with the key identified global frames and concepts of interest. All final included documents were read and then coded. Codes were added to the codebook as new themes, frames, or concepts emerged. Weekly meetings were held between researchers during the African regional policy project design, data coding, analysis, and write-up phase for sharing progress and preliminary findings. These meetings provided opportunities for critique or insights from other researchers and assisted in refining the analysis and synthesis of findings.

## 3. Results

At the global level, 62 diet-related policy documents were analysed. These documents were primarily resolutions and declaration documents from UN endorsed global assemblies and sessions such as the World Health Assembly, UN General Assembly, UN Economic and Social Council, Codex Alimentarius Commission, and FAO’s Committee on Agriculture. These documents contained annexes with endorsed global action plans and strategies, which were included in the policy analysis.

Drawing on the idea of policy framing, three frames were identified across global policy documents for describing and positioning the NCD policy problem. These were the (i) informing evidence-based frame and the interconnecting macro-level context frames of (ii) development and (iii) socioeconomics. The evidence-based frame contained ‘informing’ discourse related to research and knowledge dissemination that focused on measuring the burden of the disease and using data to identify health inequities. The development frame is related to the wide-scale impacts that globalisation and subsequent urban, technological, and industrial developments have had on health, food environments, and urban behaviour, while the socioeconomics frame addressed interactions between social and economic factors that have an impact on health and the NCD burden. Taken together, these contributed towards developing a rationale for the interconnected intervention approaches for the NCD ‘solution’, which guide policy solutions for NCDs, namely (i) health promotion, supported by (ii) intersectoral/multisectoral support, and (iii) evidence-based monitoring and assessment of interventions and progress.

**Figure 3:**
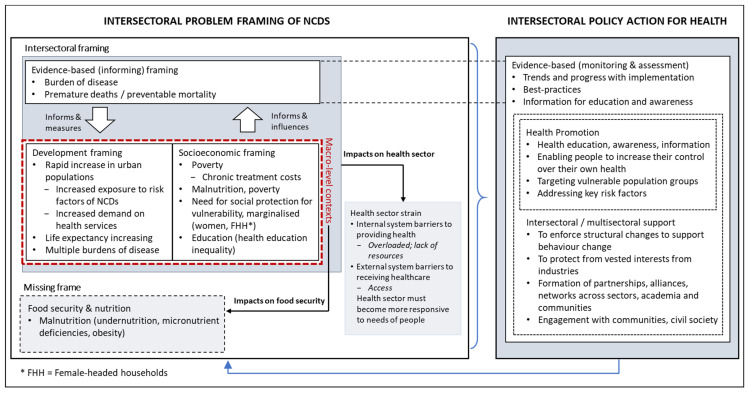
Conceptual framework of the African regional level frames that depicts two primary categories: the first (**left block**) comprising key frames related to NCD problem framing and the second (**right block**) highlighting approaches for policy action for health. Two of the policy problem frames (**left block**), namely ‘development’ and ‘socioeconomics’, are interrelated macro-level contexts. Regarding the intersectoral policy action for health (**right block**), health promotion is nested within intersectoral/multisectoral support, which are both supported by evidence-based monitoring and assessment.

At the African regional level, 29 diet-related policy documents were reviewed from various sectors relating to diet and health and which specifically mentioned or referred to NCDs. The same overarching problem and solution framings emerged at the African level, although there were some differences in themes within each frame. Figure 3 depicts the problem framing of NCDs (left block) at the regional level and the three interconnected intervention approaches contributing to solution framing for regional policy action for NCD (right block). Examples of key text for each frame were isolated from the African regional policy documents and presented in Table 2.

### 3.1. Problem Stream: Intersectoral Problem Framing of NCDs

At the global level, the three primary frames used to construct the NCD policy problem, namely, the (i) evidence-based, (ii) development, and (iii) socioeconomic frames, were classified as intersectoral as the frames were not specific to any one sector but instead represented interconnections between multiple sectors. These were also the primary frames at the African regional level. An additional regional theme of health sector strain emerged during the analysis; however, it was not classified as an NCD intersectoral problem frame as its focus was specific to the health sector. Food security was not found to be a policy frame for NCDs, although this concept was identified as contributing to NCD risk factors.

#### 3.1.1. Evidence-Based Frame

##### The Burden of Disease

Global and regional policy documents utilised aggregated data and standardised indicators to highlight the severity of the NCD burden. For example, the World Health Assembly highlighted that NCDs contributed to the majority (60%) of global deaths [26]. Similarly, the African documents drew attention to the predicted global rise in NCD from 40% of global morbidity in 1990 to an estimated 60% in 2020 [21,23]. However, the African documents predicted that sub-Saharan Africa (SSA) would experience a greater increase in NCD and injury burden compared to global trends, from 28% of morbidity in 1990 to an expected 60% in the region by 2020, if communicable disease goals are attained [23]. By 2010, 40% of deaths in the African region were attributed to NCDs [21,27,28]. These revelations contributed to a shift in global attention onto NCDs, which were previously overshadowed by a global focus on HIV, malaria, and tuberculosis [29].

##### Concepts of Premature Mortality and Preventable Mortality

Traditional epidemiological transition theory originally associated NCDs with old age [30]. However, global and African policy documents presented evidence highlighting the concept of premature mortality in that NCD-related deaths are increasingly occurring in people under 70 years—the global average life expectancy [31,32]. Related to premature mortality—although not synonymous—is preventable mortality and morbidity. Both global and African regional policies emphasised that while the burden of NCDs is high and accelerating in LMICs, many cases of NCD mortality and morbidity are preventable by utilising improved NCD case-management and addressing modifiable NCD risk factors of tobacco use, unhealthy diet, alcohol consumption, and physical inactivity [21,23,33,34].

##### The Need for Data for Health Equity Analysis

Data disaggregated by place, time, demographics, and other key characteristics can highlight underlying inequities, masked by aggregation [35,36]. Global policy documents specifically highlighted insufficient data disaggregated by age, sex, and socioeconomic status for monitoring NCDs and their risk factors in LMICs [33,37,38]. African policy documents acknowledged the need for disaggregated data; however, the narrative focused on the paucity of general NCD data for the region [23,36]. One document reported that many health facilities in Africa do not report on NCDs, resulting in underestimated disease prevalence [39]. Insufficient data and knowledge dissemination on the African NCD problem was suggested as a contributing factor for sub-optimal attention on the NCD problem and the perception of a non-existent NCD burden [23,36].

#### 3.1.2. Development Frame

A reciprocal relationship is evident between development and NCDs. Firstly, global documents emphasised the wide-scale impacts that globalisation and subsequent urban, technological, and industrial developments have had on health, food environments, and urban behaviour. Documents posited that this contributed to a nutrient transition that is especially prominent in LMICs, characterised by increased consumption of processed foods including refined sugars and carbohydrates, preservatives including salt, and saturated fats [29,40,41]. Relatedly, global policies highlighted how globalisation and urbanisation influenced trends in trading, advertising, and marketing of certain products in LMICs (e.g., fast-food, tobacco, and alcohol), which intersected with factors such as proliferating unplanned urban areas, poverty, malnutrition, high communicable disease burden, and inadequate healthcare systems that together created complex interactions that are conducive to a rise in NCDs [29,32]. African policy documents acknowledged this impact of rapid urbanisation and globalisation on nutrition and NCD trends [23,42]. What is of note is that concepts of diet or nutrition were not used as frames for NCD narratives at either level but were instead placed within discourses of urbanisation and development, especially in the context of LMICs.

Secondly, global and regional documents highlighted the impact that NCDs have on development, particularly for LMICs. NCDs were considered “*major challenges to sustainable development in the 21st Century*” [43] (p. 1), and the African Union highlighted that premature and preventable mortality influences Africa’s development progress, particularly in meeting development goals and targets [22]. Linking to the evidence-based frame, 15 global policy documents specifically referred to premature mortality within the context of sustainable development or the global development targets. At least seven global policies highlighted the impact that premature mortality has on LMIC development [31,32,44,45,46,47,48]. Likewise, preventable mortality was linked to development in global and African policies, as LMICs are less able to prevent NCD-related mortality if their health systems remain overburdened and under-resourced in terms of budget allocation, medical technology, and data and information systems. The concept of preventable mortality within the development frame provides a moral argument for effective prevention strategies to save lives and for improved resources to relieve the strain on health sectors of LMICs [23]. To develop and effectively implement prevention interventions, intersectoral collaboration and efforts led by non-health sectors will be required as the upstream determinants of health and modifiable risk factors need to be targeted.

The development framing of the NCD problem at both global and regional levels was likely driven by the omission of NCD targets and goals from the Millennium Development Goals in 2000 [27]. With the endorsement of the 2015 Sustainable Development Goals, which included NCD-related targets, there was a continuation of global and regional effort to address NCDs as part of the sustainable development agenda and a call for NCDs to be placed on the national development agendas of countries [40].

#### 3.1.3. Socioeconomic Frame

Since 2011, global and African regional policies acknowledged a complex ‘vicious cycle’ between poverty and NCDs [27,29,40,49]. Firstly, global policies described NCDs as “*major challenges for the economic and social development in the 21st Century*” [7,37]. NCDs reportedly limit economic productivity and are associated with high chronic treatment costs, thereby having socioeconomic consequences from individual to population level [29,41]. Similarly, African policies specify that “*NCDs, often occurring at ages of increased responsibility, deprive families of precious income and communities of productivity reserves*” [23] (p. 129). Moreover, the economic cost of NCDs was acknowledged at the forefront of the 2012 WHO Regional Office for Africa’s (WHO AFRO) discussion on NCDs [23]. This socioeconomic impact narrative created a sense of urgency across global and regional levels for proactive and integrated approaches that contribute to achieving sustainable socioeconomic development through health equity.

Secondly, albeit less prominent, global and regional policies implied that socioeconomic factors shape and determine NCD burden, especially as poverty influences the conditions in which people live, which are important health determinants [23,37]. Global policies highlighted that “*poverty, underdevelopment and low socio-economic status are major contributors to malnutrition in both rural and urban areas*”—a key risk factor of NCDs [50] (p. 1). Insufficient infant and child feeding practices, inadequate access to healthy foods, or food and water safety concerns were identified as determinants for malnutrition [40,41,50]. Within African policies, malnutrition was a theme expressed mostly in relation to vulnerable groups, particularly those that are poor and marginalised. This is likely due to the link between poverty and social and health inequities which together reduce opportunities to prevent NCD mortality and morbidity [24,36].

#### 3.1.4. Missing NCD Problem Frame at the African Level: Food Security

Food security or “when all people, at all times, have physical, social and economic access to sufficient, safe and nutritious food that meets their dietary needs and food preferences for an active and healthy life” [51] (p. 43) is an important concept for the African region. Despite key links to diet—a risk factor of NCDs—the dialogue around food security in regional policies generally omitted conversations relating to NCDs, thus representing a missing frame for the NCD problem. Instead, food security was identified as a determinant of health and occasionally mentioned in relation to malnutrition [51]. Malnutrition may, thus, act as a possible intermediary concept between food security and NCDs dialogues; however, this was only found within five documents from 2014 onwards [51,52,53,54,55].

It is possible that connections between food security and NCDs are acknowledged in policies that fell outside the scope of this study. To broaden this specific interrogation so as to confirm that no documents or text were overlooked during data cleaning, previously excluded African regional policies from Phase 1 (data cleaning) with any relevance to food security, diet, or nutrition were re-examined for missing links to NCDs.

The findings from the re-examination process showed that of the eight food-related documents that were previously excluded during Phase 1 due to their lack of reference made to NCDs, four were from the African Development Bank, two were from the NEPAD, one from the FAO, and one from the African Union Commission. All eight documents were explicitly related to food or agriculture, and none made any reference to NCDs. NEPAD’s [56] Comprehensive Africa Agriculture Development Programme only mentioned disease in relation to crop and livestock disease and pests. Therefore, these African agricultural and food-specific policies largely considered factors that relate to achieving food security and not the explicit impact that food security has on NCDs.

### 3.2. Policy Stream: Intersectoral Policy Action for Health

Global policies acknowledged that the prevention and control of NCDs is an emerging priority; however, it requires a paradigm shift from a sole reliance on biomedical intervention approaches, towards including collaborative approaches that emphasise participation from non-health sectors in addressing the upstream determinants of health [46]. Examples of specific interventions were gathered from regional policies. These spanned themes of data and research, advocacy and empowerment, private sector measures, institutional structures, and health sector reform. Health promotion was mentioned across interventions and contributed an underpinning ideology for addressing NCDs and improving overall health. However, global and regional policies suggested that health promotion requires support from intersectoral and multisectoral efforts, while evidence-based monitoring and assessment must guide and measure intervention implementation. This section explores how health promotion (supported through intersectoral and multisectoral efforts and guided and evaluated through evidence) was used to frame policy solution narratives around NCDs at the African regional level.

At the African regional level, at least four documents explicitly promoted and endorsed health promotion for addressing health challenges, including NCDs [21,25,57,58]. Health promotion for addressing NCDs was also mentioned in an additional six regional policies. Health promotion was defined in African policies in line with the Ottawa Charter for Health Promotion as “*a process of enabling people to increase control over, and improve, their health. Health promotion seeks to promote healthy behaviours and empower individuals, families, households and communities to take appropriate action to that end. It reinforces the desired social and structural changes through policies, legislation and regulation*” [28] (p. 40).

Overall, endorsing health promotion for addressing NCDs depicted an expansion on the traditional approach, which primarily relied on biomedical solutions (e.g., case management and treatment) [24]. By embracing health promotion, the African policy ‘solution stream’ acknowledged the preventability of NCDs—important for addressing premature mortality—which relies on addressing risk factors such as tobacco, excessive alcohol use, physical inactivity and unhealthy diet. Key components of NCD risk factor interventions (which cut across framings of health promotion, intersectoral/multisectoral action, and evidence for monitoring and assessment) included (i) education and awareness, (ii) improving data for monitoring and surveillance of risk factors, and (iii) key structural policies, regulations, and levies, especially for alcohol and tobacco [23].

#### 3.2.1. Interventions of Education and Awareness

The term ‘lifestyle’ featured prominently in both global and regional policy documents, including language such as ‘lifestyle diseases’ [31] and ‘lifestyle-related’ or ‘behavioural’ risk factors [32,47,58]. Relatedly, a narrative around interventions for education and awareness implied the perception that human behaviour and choice influence exposure to risk factors and can either support or hinder wider action to improve structural characteristics in environments. Within regional policies, interventions for empowerment through education, information dissemination, and awareness sought to increase the following: (i) understanding of risk factors and exposures, (ii) community support for intersectoral and multisectoral efforts to limit exposure to risk factors, and (iii) community participation in problem identification and in the design and implementation of interventions [23].

“*Provision of information for decision-makers and communities should be strengthened in order to increase commitment to public health protection, recognition of alcohol-related harm in the community and active participation in policy measures and in implementation.*”

[23] (p. 208)

#### 3.2.2. Interventions for Data, Evidence-Based Monitoring, and Surveillance

Interventions for improving data for NCD monitoring and surveillance encompassed themes such as (i) measuring the burden of disease attributable to NCDs, (ii) monitoring risk factors (e.g., alcohol consumption and production), (iii) monitoring implementation and impact of risk factor interventions, (iv) documenting structural changes, and (v) developing operational research [23]. This will involve integrating data across sectors. Interventions to improve integrated data (including data able to be disaggregated by time, place and other characteristics) and NCD surveillance will contribute to addressing the paucity of NCD-relevant data for the African region [59]. This would also assist in identifying vulnerable groups and inequities in health.

“*It is important to have local data on the disease burden attributable to NCDs, their risk factors and major determinants. This knowledge will facilitate priority setting and adoption of appropriate actions. Wherever data are limited, specific baseline studies should be conducted. Evidence strengthens advocacy and facilitates decision-making.*”

[36] (p. 5)

#### 3.2.3. Interventions for Structural Changes

Interventions that seek to support structural changes through legislation, policies, and regulations imply a recognition that healthy human behaviour may be confined or influenced by structural factors in the urban system or environment. The most apparent interventions in this regard are related to the development of regulations, levies, and legislation to protect consumers and limit exposure to alcohol and tobacco smoke [23]. For example, the ‘Reduction of the harmful use of alcohol: A Strategy for the WHO African Region (AFR/RC60/4)’ encouraged national alcohol taxes, and ‘Cancer prevention and control: A strategy for the WHO African Region’ sets targets for reducing tobacco smoke exposure and encourages legislation and regulations for this [23].

A vague narrative remained around nutrition-specific or food-specific interventions or related approaches for purposefully addressing NCDs through African food environments. Specifically, very little direction was given to non-health sectors on structural interventions for addressing NCDs through food environments. Only one intervention theme was found in this regard and was related to food regulations and legislation for the protection of individuals, communities and families from unhealthy diets, and to protect health policies from the vested interests of the food industry [21,49]. However, only one document acknowledged that food industry regulations can be used to specifically address the wider NCD challenge [49]. Moreover, regional policies addressing NCDs only mentioned nutrition cursorily in the context of health interventions, with only four documents mentioning nutrition in their intervention recommendations. Nutrition, diet, or food-specific interventions for purposefully addressing the NCD burden were generally deficient.

Instead, in the regional nutrition-specific policy documents, such as the ‘African Regional Nutrition Strategy 2015–2025’ [53], the focus of diet, nutrition, and food-related interventions was on addressing malnutrition, which includes undernutrition, overnutrition, and micronutrient deficiencies. While nutrition-specific or malnutrition-related interventions would inevitably contribute to addressing NCDs, this link is generally superficially implied and understated. For example, the African Regional Nutrition Strategy presented details on key recommendations to improve nutrition; however, these were framed external to any dialogue on NCDs and were recommended for implementation through agricultural programmes [53]. The impact of such interventions on the overall NCD burden is never mentioned or encouraged to be scrutinised, monitored, or evaluated.

## 4. Discussion

This analysis of diet-related global and African regional policy documents relevant to food, nutrition, and NCDs provides important insights into the conceptualisation of the NCD problem, which can be discussed through three points. Firstly, the use and interpretation of data and evidence-based information are important for problem framing and for informing and developing interventions. Secondly, the concept of preventability was a recurring theme across problem framings of NCDs. Finally, the use of an alternative framing approach allows exploring the amenability of diet-related policies for intersectoral action to address NCDs.

### 4.1. The Role and Influence of Aggregated and Disaggregated Data in Policy Documents

The findings highlighted that both disaggregated and aggregated data are essential for identifying, exploring, and monitoring the extent of the NCD problem, including the systemic factors of the built and food environments contributing to the burden of disease and the inequities present across population group and geographic place. These data guide the development and implementation of context-relevant interventions, including those related to education and awareness. Aggregated health data are useful for measuring progress in achieving global targets as a form of accountability. Standardised indicators and measurement are, thus, important for accurate monitoring and evaluation processes. Insufficient local and national data collection on the NCD burden and associated determinants in many African LMICs may continue to hinder the region’s progress in implementing NCD policies and strategies [60]. Within global and regional policies, narratives around evidence-based concepts of premature and preventable mortality were used to motivate action and exemplifies how data may not always be used impartially but can be used to reinforce a particular frame or perspective and justify changes in policy agendas. This analysis supports the notion that policy agendas are socially constructed and, thus, can be affected by bias [2]. Therefore, while data provides important evidence, their use and interpretation must be met with scrutiny.

### 4.2. The Concept of Preventability of NCDs

Global and regional framing of the NCD problem emphasised the concept of prevention in relation to premature mortality. This supports the finding of Whyte [61], which report that the WHO has a primary interest in initiatives to prevent NCDs by targeting risk factors. NCDs have previously and controversially been referred to as ‘lifestyle diseases’ due to associations with risk factors such as diet, alcohol, tobacco use, and physical inactivity [61]. However, the ‘lifestyle disease’ perception detracts from the inequities experienced by population groups and countries, as well as the influence that wider structural factors in the built and food environments have in shaping behaviour and health. This is supported by Oni’s [62] categorisation of the eight S’s of urban exposure that draw attention to the influence that urban built and food environments have in shaping health. As the socioeconomics and development frames both acknowledge the influence that socioeconomic and development factors have on health, these may be used as a rationale for supporting the development of intersectoral interventions that address broader structural factors that shape health outcomes.

### 4.3. The Amenability of Diet-Related Policies for Intersectoral Action to Address NCDs

The framing of NCDs within current diet-related policies in Africa is conducive for intersectoral action agendas for health, which should focus on empowerment and supporting social and structural changes through policies, legislation, and regulations. A few African regional policy issues must be considered and addressed in order to create a more amenable policy environment for effective intersectoral action in addressing NCDs.

Firstly, while regional policy documents provide guidance on various types of interventions for addressing NCDs, the link between food and nutrition-specific interventions and NCDs is insufficiently acknowledged. As the concept of ‘unhealthy diet’ is one of the four most promoted risk factors of NCDs, it is concerning that African diet-related policies do not acknowledge the wider impact that food, nutrition, and agricultural interventions have on the NCD burden, which has been described as a serious public health, socioeconomic, and development concern for Africa. Findings revealed that the agricultural sector in Africa was given responsibility for overseeing interventions to address malnutrition and food security, which were encouraged to be conducted in collaboration with other sectors and programmes. However, due to the lack of explicit acknowledgement of the link between food, diets, and NCDs, there is a risk that a collaborative partnership between the agricultural sector and the health sector may be viewed as redundant. While diet-related interventions are likely to inadvertently result in health improvements, a failure to consider the impact of food environment interventions on the NCD burden denies opportunity to monitor the impact of interventions on the burden of disease and associated risk factors and, thus, opportunity for accountability. While the scope of this study did not include all food-relevant and diet-relevant policies, sufficient evidence was available to suggest that African agricultural and food-specific policies largely do not contain considerations for (i) the impact that food security has on current burdens of NCD and (ii) the impact of food environment interventions on NCDs and associated risk factors.

Secondly and relatedly, a disjuncture may form between the development frame of the NCD problem and the proposed interventions of the NCD solution stream, particularly at the African regional level where development is a large concern. Specifically, the development frame highlighted the large impact that structural macro-level factors such as globalisation and urbanisation may have on shaping the built and food environments, which in turn impacts behaviour or human agency. However, within the narrative around NCD-specific interventions at the regional level, insufficient attention was given to addressing structural factors relating to the built and food environment. While African policies acknowledged the need for both social and structural changes, a greater emphasis was placed on education and awareness when providing guidance on NCD interventions, and only vague direction was provided for non-health sectors in which urban macro-level components need addressing. Therefore, there is a possibility that future regional policies may place increasing responsibility on individuals and communities to adopt healthy behaviours while overlooking the health-impairing components of built and food environments that lie outside the control of individuals and communities.

Thirdly, while NCD problem frames acknowledged an interrelation between health, development, and socioeconomic wellbeing, a more explicit narrative was placed on ‘health for development,’ as NCDs were described as major challenges to economic, social, and sustainable development. One regional policy document stated that “*the ultimate outcome of effective health promotion interventions is a healthy and productive generation […] that leads to improved social and economic development*” [21] (p. 1). Insufficient acknowledgement by non-health sectors of the impact of built and food environment structural interventions on NCDs suggests that the interrelation between development and health is at risk of becoming asymmetrical in which ‘development for health’ is overlooked. To effectively address the burden of disease attributable to NCDs, considerations for the health impacts of interventions must be at the forefront of non-health sector intervention development. Development for health is especially important for the African region due to the overloaded health sector.

## 5. Conclusions

This study demonstrated the use of an alternative framing approach for exploring the problem framing of NCDs within diet-related policies at global and African regional levels, as well as the interrelation between the problem framing and proposed policy interventions. There is a complex relationship between NCDs and socioeconomic and development processes. These interactions should be considered as far as possible within agendas for intersectoral action to address NCDs and highlight the need for improved data collection and disease surveillance, particularly for LMICs. As the health sector in the African context is reportedly strained, solutions that take the pressure off the health sector and those that are driven by non-health sectors are particularly important not only for addressing the NCD burden but also for development and economic wellbeing. Intersectoral strategies should seek to support social and structural changes in the built and food environments through strategic planning, policies, and regulations. As the African regional policy documents largely overlooked the link between food security and NCDs, an opportunity is presented for the inclusion of NCD considerations into the food security agenda for addressing NCDs through diet and nutrition at national levels. Malnutrition was identified as a possible intermediary concept for linking food security to NCD narratives and may, thus, be used to motivate for considerations for intersectoral interventions that improve access to nutritious food for preventing NCDs. Data must be used to monitor impacts of interventions on the local burden of disease.

## Figures and Tables

**Table 1:** Search terms used to explore diet-related policy documents relevant to NCDs.

Primary Term	Abbrev.	Key Term of Interest	Alternative Terms	Sub-Terms (Internal Search)
Non-communicable disease	NCD(s)	NCD(s)	Non-communicable, noncommunicable	Diabetes, hypertension
Diet			Nutrition, food	Sugar, salt
		Sugar	Sugars, glucose, sugar-sweetened	Nutrition, diet, food
		Salt	Sodium	Nutrition, diet, food

**Table 2:** Samples of African region policy text provided for each of the identified frames for NCD problem and solution streams.

		Framing	Text Example	Key Words
Problem framing of NCDs	Macro-level context	Evidence-based (informing and research)	“According to the WHO Global Burden of Diseases Report (2008), this burden accounted for a total of 58.8 million deaths worldwide in 2004 and 18.6% of the deaths were in the WHO African Region” [21] (p. 1).	Mortality, morbidity, preventable, and premature deaths
	Macro-level context	Development	“The triple burden from communicable and non-communicable diseases and injury and trauma, including the social impact of these, has adversely affected development in Africa. Africa is still not on track to meet the health Millennium Declaration targets and the prevailing population trends could undermine progress made” [22] (p. 3).	Development, development goals, life expectancy, and urbanisation
	Macro-level context	Socioeconomics	“NCDs, often occurring at ages of increased responsibility, deprive families of precious income and communities of productivity reserves” [23] (p. 129).	Poverty, vulnerable populations, and education
Solution framing for addressing NCDs	Data approach	Evidence-based (monitoring and assessment)	“Strong monitoring and evaluation mechanism. To ensure that the set objectives of the complex interventions are met, it is necessary to measure improvement, efficacy and efficiency as well as qualitative aspects such as equity, fairness, gender sensitivity and community involvement” [24] (p. 4).	Measure, efficiency, and technical support
	Intervention approach	Health promotion (nested within intersectoral/multisectoral support)	“The health promotion interventions proposed for the African Region are based on multisectoral approaches to tackling priority public health conditions. They address the preventable causes of disease, disability and premature deaths in the Region in all population groups throughout the life course” [21] (p. 6).	Life-course, community health awareness, participation, empowerment
	Governance approach	Intersectoral/multisectoral support	”To establish, as appropriate, multisectoral and interministerial mechanisms for promoting health through Health-in-All-Policies, good governance for health, community participation, social dialogue, partnership and leadership/stewardship roles” [25] (p. 2).	Multisectoral, intersectoral, and interministerial, whole-of-government

## Data Availability

Not applicable.

## Supplementary Materials

The following are available online at https://www.mdpi.com/article/10.3390/ijerph182111246/s1, Table S1. Historical timeline mapping of African regional policies related to diet and/or noncommunicable disease.
