# Supplementary material for: Intersectoral Action for Addressing NCDs through the Food Environment: An Analysis of NCD Framing in Global Policies and Its Relevance for the African Context
Source: Int J Environ Res Public Health. 2021 Oct 26;18(21):11246. doi: 10.3390/ijerph182111246 (PMC8582825; doi:10.3390/ijerph182111246)
Supplement: Supplementary file 1 [file ijerph-18-11246-s001.zip › ijerph-1399381-supplementary.pdf]

Table S1. Historical timeline mapping of African regional policies related to diet and/or noncommunicable disease

HISTORICAL TIMELINES - REGIONAL POLICIES									INCLUDED (29)	Food security/ nutrition security docs (previously excluded) to check for NCD link (9)	Additional documents considered (55)	Not official document (event; programme)
		2000	2001	2002	2003	2004	2005	2006	2007	2008	2009
Documents - List of docs (Document name)	1) Doc 1	African Union (AU) 2000 Thirty-Sixth Ordinary Session	Snowball: WHO AFRO. 2001. Regional Committee for Africa Fifty-first session Report of the Regional Director on Health Promotion: A strategy for the African Region	AU 2002 First Ordinary Session	New Partnership for Africa's Development (NEPAD). 2003. Comprehensive Africa Agriculture Development Programme (CAADP). (Maputo Declaration)	AU 2004 Third Ordinary Session Union	AU 2005 Fifth Ordinary Session	AU 2006 Seventh Ordinary Session	AU 2007 Eighth Ordinary Session	AU 2008 Eleventh Ordinary Session - adopted AHS 2007-2015	AU 2009 Thirteenth Ordinary Session
	2) Doc 2	Snowball: WHO AFRO. 2000. Regional Committee for Africa Fiftieth session Report of the Regional Director on Noncommunicable Diseases: A Strategy for the African Region		AU 2002 Second Extraordinary Session	AU 2003 Second Ordinary Session		AU 2005 Fourth Extraordinary Session	AU 2006 Sixth Ordinary Session	AU 2007 ninth Ordinary Session	AU 2008 Tenth Ordinary Session	AU 2009 Twelifth Ordinary Session
	3) Doc 3	Snowball: WHO AFRO. 2000. Regional Committee for Africa Fiftieth session Resolution on Noncommunicable Diseases: A Strategy for the African Region		Organization of African Unity. 2002. Assembly of Heads of State and Government Thirty-Eighth Ordinary Session of the Organization of African Unity: Decisions and Declarations			AU 2005 Fourth Ordinary Session	WHO AFRO. 2006. Regional Committee for Africa Fifty-sixth session Round Table on Intersectoral Action for Health Promotion and Disease Prevention	Snowball: African Union. 2007. Third session of the African Union Conference of Ministers of Health: African Health Strategy 2007-2015	Snowball: Ouagadougou Declaration 2008 (endorsed by Regional Health Committee in 2008) REF: REF: WHO 2010 Achieving Sustainable Health Development	AFDB 2009 Gender mainstreaming checklist for the health sector
	4) Doc 4	African Development Bank. 2000. Agriculture and Rural Development Sector Bank Group Policy		Snowball: WHO AFRO. 2002. Regional Committee for Africa Fifty-second session Poverty and Health: A Strategy for the African Region			African Union. 2005. African Regional Nutritional Strategy 2005-2015		REF: WHO AFRO. 2007. Regional Committee for Africa Fifty-seventh session Report of the Regional Director on Diabetes Prevention and Control: A Strategy for the WHO African Region	REF: WHO AFRO. 2008. Libreville Declaration on Health and Environment in Africa. WHO AFRO: Libreville	International Conference on Diabetes and Associated Diseases. 2009. The Mauritius Call For Action
	5) Doc 5								REF: AFR/RC57/R4 Resolution - Diabetes prevention and control: a strategy for the WHO African Region - included in above	African Development Bank Group. 2008. The African Food Crisis Response (AFCR)	WHO AFRO. 2009. Regional Committee for Africa Fifty-ninth session Policy orientations on the establishment of centres of excellence for disease surveillance
	6) Doc 6										
	7) Doc 7										
	8) Doc 8										
	9) Doc 9										
	10) Doc 10										
	11) Doc 11										

	INCLUDED (29)	Food security/ nutrition security docs (previously excluded) to check for NCD link (9)	Additional documents considered (55)	Not official document (event; programme)					
2010	2011	2012	2013	2014	2015	2016	2017	2018	2019
REF (NEPAD) First Africa Day for Food and Nutrition Security	REF (NEPAD) CAADP Nutrition Initiative endorsed to mainstream nutrition in National Agriculture and Food Security Investment Plans)	AU 2012 Eighteenth Ordinary Session	REF: Accelerating Nutrition Improvements (ANI) project for SSA commenced from 2013 - 2016	REF (NEPAD): African Union launches 2014 Year of Agriculture and Food Security	REF: World Health Assembly Resolution "calling on Member States to report biennially on progress being made towards the global nutrition targets for 2025"	REF (NEPAD): UN Declares Decade of Action on Nutrition 2016-2025	AU 2017 Twenty-Eight Ordinary Session	AU 2018 10th Extraordinary Session of the Assembly	AU 2019 12th Extraordinary Session of the Assembly
AU 2010 Fifteenth Ordinary Session	AU 2011 Seventeenth Ordinary Session	AU 2012 Nineteenth Ordinary Session	AU 2013 Extraordinary Session of the Assembly	REF (NEPAD): SADC Ministers for Agriculture and Food Security and Ministers of Health agree on strategies to increase food production	AU 2015 Twenty-Fifth Ordinary Session	AU 2016 Twenty-Seven Ordinary Session	AU 2017 Twenty-Ninth Ordinary Session	AU 2018 11th Extraordinary Session of the Assembly	AU 2019 First Mid-Year Coordination Meeting
AU 2010 Fourteenth Ordinary Session	AU 2011 Sixteenth Ordinary Session	WHO AFRO. 2012. Compendium of Public Health Strategies Volume 1	AU 2013 Twentieth Ordinary Session	AU 2014 Twenty-Second Ordinary Session	AU 2015 Twenty-Fourth Ordinary Session	AU 2016 Twenty-Sixth Ordinary Session	New Partnership for Africa's Development (NEPAD). 2017. The Comprehensive Africa Agriculture Development Programme: Programmatic and institutional overview	AU 2018 Thirtieth Ordinary Session	AU 2019 Thirty Second Ordinary Session
Food and Agriculture Organization. 2010. FAO Regional Strategic Framework for Africa 2010-2015.	Snowball: WHO AFRO. 2011. The Brazzaville Declaration on Noncommunicable Diseases Prevention and Control in the Who African Region	WHO 2012 Report of the Commission on Women's Health	AU 2013 Twenty-First Ordinary Session	AU 2014 Twenty-Third Ordinary Session	Southern African Development Community. 2015. Food and nutrition security strategy 2015-2025	African Union Department of Social Affairs. 2016. Africa Health Strategy 2016 – 2030	African Development Bank Group. 2017. "Say No To Famine" Framework Document (Revised Version)	AU 2018 Thirty-First Ordinary Session	69th WHO AFRO Regional Committee Meeting (NCD Alliance Advocacy Briefing)
WHO AFRO. 2010. Achieving Sustainable Health Development in the African Region: Strategic Directions for WHO 2010-2015		WHO AFRO Regional Committee for Africa. 2012. Regional Committee for Africa Sixty-second session Draft for Discussion on WHO General Programme of Work	WHO Regional Director's closing remarks	African Union Commission. 2014. Malabo Declaration on Accelerated Agricultural Growth and Transformation for Shared Prosperity and Improved Livelihoods.	AU 2015 Agenda 2063 The African We Want	African Development Bank. 2016. Feed Africa: Strategy for Agricultural transformation in Africa	WHO 2017 Leave no one behind strengthening health systems	New Partnership for Africa's Development (NEPAD). 2018. Pan African Parliament Resolution on Nutrition and Food Systems in Africa	NCDA Joint Statement Agenda Item 14 NCDs (AFR/RC69/10)
		2012 AFR-RC62-12-implementation-of-international-health-re		African Union and World Health Organization. 2014. First meeting of African Ministers of Health jointly convened by the AUC and WHO	AU 2015 Agenda 2063 Implementation Plan				AFDB 2019 East Africa Regional Strategy 2018-2022
		WHO AFRO. 2012. Regional Committee for Africa Sixty-second session Resolution on The African Health Observatory: Opportunity for Strengthening Health Information Systems Through National Health Observatories			WHO 2015 The Africa Health Transformation Programme 2015-2020				WHO 2019 The transformation agenda of the WHO secretariat in AFRO, 2015-2020, Taking Stock; Consolidating and Stepping-Up: January 2019-January 2020
		WHO AFRO. 2012. Regional Committee for Africa Sixty-second session Resolution on Health Promotion: Strategy for the African Region			WHO AFRO. 2015. Regional Committee for Africa Sixty-fifth session Report of the Secretariat on Progress Report on the Establishment of the Africa Centre for Disease Control				WHO Africa. 2019. Regional Committee for Africa Sixty-ninth session Draft Resolution on Strategic Plan to Reduce the Double Burden of Malnutrition in the African Region
		WHO Africa. 2012. Regional Committee for Africa Sixty-second session final report			African Union Commission. 2015. The Africa Regional Nutrition Strategy 2015-2025				WHO AFRO. 2019. Regional Committee for Africa Sixty-ninth session Report of the Secretariat on Strategic plan to reduce the double burden of malnutrition in the African Region: 2019-2025
		WHO AFRO. 2012. Regional Committee for Africa Sixty-second session Consideration and Endorsement of the Brazzaville Declaration on NCDs							WHO AFRO. 2019. Regional Committee for Africa Sixty-ninth session Draft Report
		WHO AFRO. 2012. Regional Committee for Africa Sixty-second session Report of the Secretariat on Health Promotion: Strategy for the African Region							2019 AFR-RC69-6 Regional Strategy for IDSR 2020-2030 - see report
